# Design and application of coal gangue sorting system based on deep learning

**DOI:** 10.1038/s41598-024-67323-z

**Published:** 2024-07-17

**Authors:** Kun Zhang, Zhen Wang, Zengbao Zhang, Zhiyuan Shi, Yuhao Qi, Mingchao Du, Yong Chen, Tijun Liu, Yumeng Chen, Zhuang Yin

**Affiliations:** 1 Shandong Provincial Key Laboratory of Robotics and Intelligent Technology, Shandong University of Science and Technology, Qianwangang Road 579, Qingdao, 266590 Shandong China; 2 Equipment Management Center, Yankuang Energy Group Co., Ltd, Kuangjiandong Road 1085, Jining, 273500 Shandong China; 3 Qinghai Energy Development Group Co., Ltd, Kuangshandong Road, Xining, 810000 Qinghai China; 4 China Mining Products Safety Approval and Certification Center, Qingniangou 5, Beijing, 100013 China; 5 Yankuang Energy Group Co., Ltd, Jizou Road, Jining No. 2 Coal Mine, Jining, 272071 Shandong China

**Keywords:** Coal gangue, Gangue sorting, Gangue detection, Deep learning, System applications, Engineering, Techniques and instrumentation, Coal, Computer science, Software

## Abstract

With the advancement of science and technology, coal-washing plants are transitioning to intelligent, information-based, and professional sorting systems. This shift accelerates the construction a modern economic system characterized by green and low-carbon development, thereby promoting the high-quality advancement of the coal industry. Traditional manual gangue picking and multi-axis robotic arm gangue selection currently suffer from low recognition accuracy, slow sorting efficiency, and high worker labor intensity. This paper proposes a deep learning-based, non-contact gangue recognition and pneumatic intelligent sorting system. The system constructs a dynamic database containing key feature information such as the target gangue's contour, quality, and center of mass. The system elucidates the relationships between ejection speed, mass, volume, angle of incidence, and the impact energy matching mechanism. Demonstration experiments using the system prototype for coal gangue sorting reveal that, compared to existing robotic arm sorting methods in coal washing plants, this system achieves a gangue identification accuracy exceeding 97%, a sorting rate above 91%, and a separation time of less than 3 s from identification to separation, thereby effectively enhancing raw coal purity.

## Introduction

Coal remains the primary source of energy in China, with its consumption consistently exceeding 50%. However, the processes of coal mining and processing inevitably produce a certain amount of coal gangue. A high gangue content not only diminishes coal quality but also lowers combustion efficiency, resulting in significant environmental pollution^1^. Typically, it is necessary to separate the gangue and transport it to the surface. A more efficient approach involves utilizing gangue in technologies for reinforcing excavations in artificial support structures^2,3^. Neglecting to treat gangue not only decreases the clean coal yield but also poses significant health and environmental hazards due to the harmful substances released during combustion^4^. Therefore, the identification, localization, and treatment of coal gangue are crucial for enhancing overall coal quality and minimizing resource waste in secondary processing. This paper presents a deep learning-based coal gangue sorting system for practical applications, enabling automatic and precise detection of gangue during the coal washing process, which is essential for achieving green coal production and intelligent coal mining.

Traditional technologies for coal gangue identification and sorting are characterized by high labor intensity, complex processes, environmental pollution, resource wastage, low sorting efficiency, and poor accuracy, which are incompatible with the demands of smart coal mining and the establishment of intelligent coal mines^5–7^. With advancements in science and technology, traditional manual selection methods are gradually being replaced by mechanized and intelligent gangue selection methods. Mechanized gangue selection is primarily categorized into jigging selection^8^, dense medium selection^9^, and dry selection methods^10^. Due to the diverse coal heavy fiber types in China and the significant compositional differences among them, the versatility of jigging selection is limited. Dense medium separation requires substantial water resources, while the dry separation method involves drying treatment, significantly increasing production costs. Intelligent gangue selection is primarily divided into ray selection and image selection. Ray-based gangue selection is mainly divided into two categories: X-ray^11^ and γ-ray^12^, which distinguish coal from gangue based on radiation attenuation levels. Image-based gangue selection is trained and optimized using deep learning algorithms, enabling the identification of coal and gangue. A comprehensive comparison of the primary gangue selection methods mentioned above reveals their respective strengths and weaknesses, as summarized in Table 1.

**Table 1:** Comparison of the advantages and disadvantages of gangue selection methods.

Gangue selection method	Main techniques	Advantages	Disadvantages
Artificial selection of gangue	Picking gangue according to the experience of workers	Simple to operate and easy to implement	High labour intensity, poor working environment and low efficiency of workers
Mechanical gangue selection	Jigging gangue selection、dense medium gangue selection and dry selection method	Simple process, high sorting efficiency	Waste of resources, high levels of pollution, poor general is ability
Ray gangue selection	X-ray and γ-ray	High degree of intelligence and sorting precision	High cost of equipment, radiation, large footprint
Image gangue selection	Deep learning, machine learning	High degree of intelligence, simple process, low production cost	Poor dynamic adaptability of online recognition and related theories need to be upgraded

In recent years, advancements in computer technology and deep learning image recognition, alongside improvements in high-definition camera performance and cost reduction, have made image sorting the mainstream technology for intelligent coal gangue sorting. Numerous scholars have conducted extensive research on intelligent sorting. Murad Saleh Alcarraza^13^ trained a coal gangue recognition model based on CGR-CNN using thermal images of coal and coal gangue. Li et al.^14^ proposed a deep learning-based hierarchical detection framework to identify coal gangue. Pengcheng Yan^15^ proposed an intelligent classification method for coal gangue using multispectral imaging technology and object detection. This method achieved a static recognition accuracy of 98.34% by enhancing the YOLOv5 model structure. Lei Zhang^16^ conducted coal gangue recognition by extracting grayscale and texture features from coal gangue images. However, there is a lack of multi-scene coal gangue datasets, the detection process is affected by noise and motion blur, image enhancement is required, and rapid sorting methods necessitate further research. The accuracy and efficiency of coal gangue recognition algorithms require further enhancement. Based on the aforementioned intelligent recognition methods, the primary sorting methods include pneumatic nozzles and multi-axis robotic arms. Yuan Huaxin^17^, Dou Dongyang^18^, and Zixiang Wang et al.^19^ proposed a jet-type coal and gangue sorting device that utilizes a camera to obtain the postural information of coal and gangue and employs an array of pneumatic nozzles for sorting. Chenguang Yang^20^ and Viljoen Jacob^21^ used X-rays to identify coal and gangue, achieving separation with high-pressure nozzles. The aforementioned non-contact pneumatic sorting methods do not require additional media and are non-polluting. However, due to variations in the size and shape of the gangue on the conveyor belt, precise control of the number and intensity of air nozzles is necessary. Small air quantities fail to achieve complete separation of gangue, whereas large quantities can affect surrounding raw coal, leading to resource waste and complex control conditions. Liu Peng^22^, Wang Peng^23^, and Junhao Jiang^24^ proposed a coal and gangue identification method based on image processing and multi-layer perceptrons and employed it to develop a coal and gangue sorting robot. Cao^25,26^ and Hongwei Ma^27^ proposed a global multi-manipulator multi-objective collaborative sorting system to address the issue of low sorting rates caused by limited space and poor coordination of single manipulators. Shang et al.^28,29^ designed a coal gangue sorting system that integrates image processing with Delta parallel robots. Compared with serial robots, its sorting speed and efficiency are significantly improved. However, with the increase in coal mining volume and intensity, the size of gangue varies, the conveyor belt speed is high, and the transportation volume is substantial. It is challenging to use mechanical arms to achieve rapid and accurate separation of gangue on high-speed conveyor belts.

In conclusion, this paper presents a deep learning-based array aerodynamic ejection coal gangue sorting system. By incorporating belt speed information, dynamic information models for the position and status of coal gangue with particle sizes of 50–120 mm are obtained. Various sorting strategies for coal gangue of differing shapes and sizes in various positions are formulated. Additionally, a dynamic motion model for varying ejection velocities, masses, volumes, and impact angles of foreign objects is established. Furthermore, the relationship between the impact energy of ejection mechanisms and gangue size and posture matching is determined. Compared to sorting robots and pneumatic nozzles, the pneumatic sorting device proposed in this paper exhibits fast response times, low energy consumption, and high sorting reliability.

## Design of coal gangue sorting system

**Figure 1:**
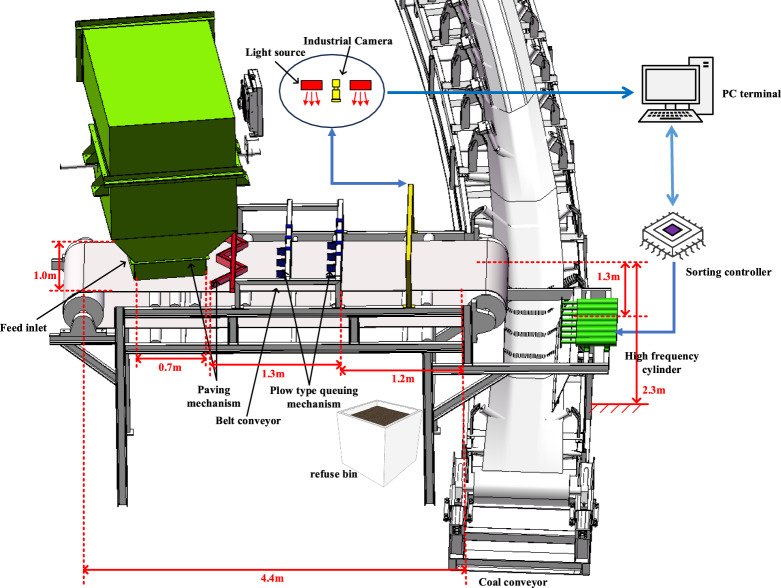
Structure diagram of gangue sorting system.

The schematic diagram of the deep learning-based coal gangue sorting system is illustrated in Fig. 1.

The primary mechanical component of the equipment is a belt conveyor measuring 4.4 m in length, 1 m in width, and 2.3 m in height. The feeding port is 0.7 m long, the paving queuing module is 1.3 m long, the coal gangue identification module is 1.2 m long, the belt conveyor's height from the ground is 2.3 m, and a 1.3-m sorting space is reserved.

**Figure 2:**
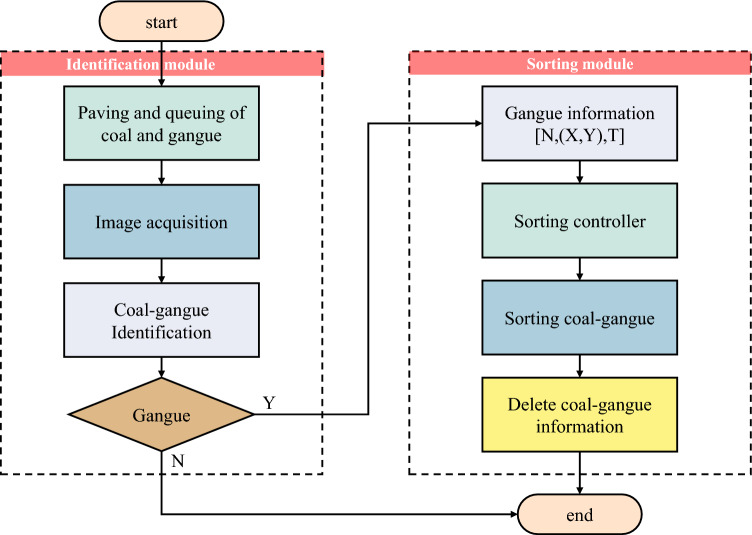
Sorting system work flow chart.

The workflow of the sorting system is depicted in Fig. 2. Where [N, (X, Y), T] is defined as follows: N denotes the number of middling coal gangue particles in the image, (X, Y) specifies the horizontal and vertical coordinates of the middling coal gangue in the image, and T indicates the recognition time of the coal gangue.

## Coal gangue recognition module based on deep learning

### Hardware design

After passing through the leveling and queuing module, coal and coal gangue enter the coal gangue recognition module to undergo the process of capturing and identifying coal gangue. The coal gangue recognition module is primarily divided into two sections: the coal gangue image capturing section and the PC terminal image processing section. The coal gangue image capture section comprises a cubic darkroom equipped with an industrial-grade digital camera array and a highly uniform strip light source. The specific equipment parameters are detailed in Table 2.

**Table 2:** Main technical parameters of some equipment in the coal gangue identification module.

Model	Parameter	Numerical value
MER2-502-79U3M/C digital camera	Interface	USB3.0
	Pixel	5 million
	Resolution ratio	2448(H)*2048(V)
	Frame rate	79.1 fps
	Exposure time	20–1000 us
DHK-TL100030 light source	Luminescent length	1000 mm
	Luminous width	30 mm
	Color	White
KPD-4A2C-24VLight source controller	Number of output channels	2
	Total output power	48W
	Output current	0–1000 mA
	Voltage range	88-264VAC
	Adjusting series	Level 256

### Coal gangue recognition algorithm based on deep learning

**Figure 3:**
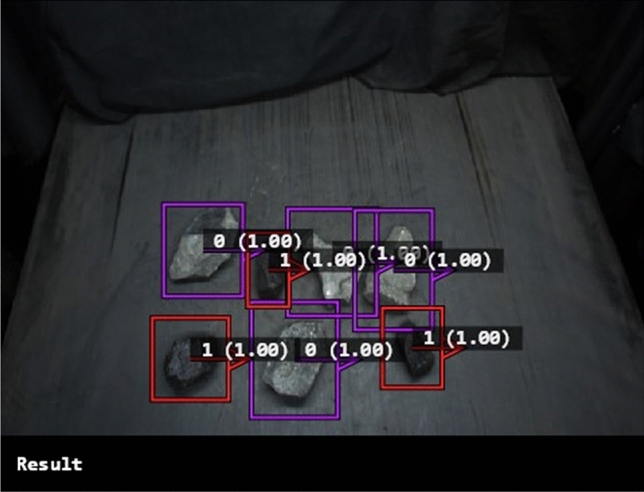
ResNet-50 network model for detecting coal gangue.

This study applies transfer learning^30^ to deep learning algorithms, selecting the ResNet-50 network, which demonstrated superior performance in HALCON software, as the coal gangue recognition module for the sorting system. The hyperparameter settings of the optimized coal gangue identification model trained on the ResNet-50 network are presented in Table 3. Figure 3. illustrates the performance of the top-performing coal gangue recognition model on various images from the test set. In the figure, the red detection box indicates the detected coal, whereas the purple detection box signifies the detected coal gangue. If coal and gangue blocks are fused, the system determines the classification based on the proportion of gangue present on the coal block as captured by the camera image. If the proportion of gangue within a coal block exceeds 50%, it is typically classified as gangue.

**Table 3:** Optimal performance ResNet-50 network model hyperparameter settings.

Name	Parameter
Batch size	2
Epoch	300
Learning rate	0.0005

### Framework for identifying coal gangue

**Figure 4:**
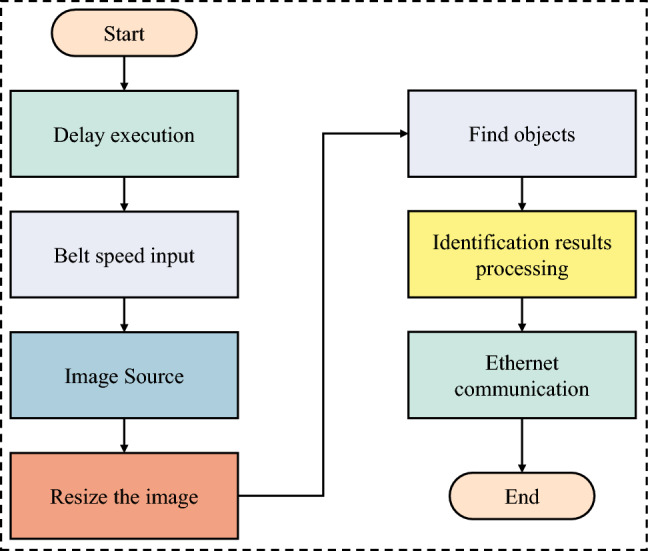
Coal gangue identification application framework workflow.

**Figure 5:**
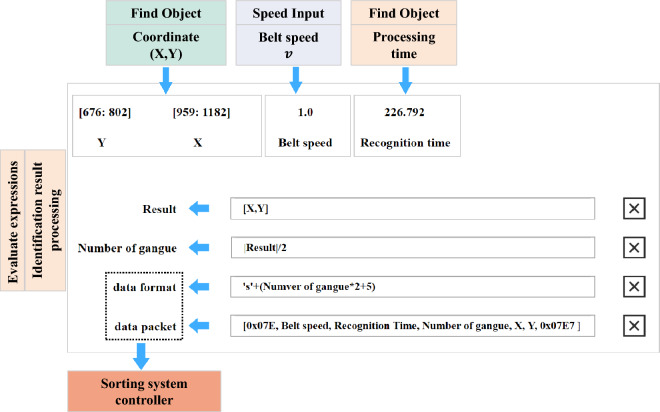
Ethernet communication.

1. Development of an application framework for coal gangue identification. The comprehensive application framework of the coal gangue recognition model based on MERLIC is illustrated in Fig. 4. It primarily consists of a speed input function module, a delay execution function module, an image source function module, an image size adjustment function module, a search object function module, a recognition result processing function module, and an Ethernet communication function module. The general workflow involves setting the photography interval based on the speed of the belt conveyor, inputting the captured images of coal and gangue into the recognition model, and subsequently transmitting the identified coal gangue information to the sorting module via Ethernet using a custom communication protocol.
2. Ethernet Communication: Process the X-axis coordinates, Y-axis coordinates, and processing time data of the identified coal gangue, and transmit the identification information packet to the sorting system controller via Ethernet. The data set is sent to the sorting system controller, as illustrated in Fig. 5.
3. Human–Computer Interaction Interface: The MERLIC Designer component in MERLIC software facilitates the creation of graphical user interfaces for machine vision applications, allowing for the insertion of controls through drag-and-drop interaction and the adjustment of layout and control properties as needed.

**Figure 6:**
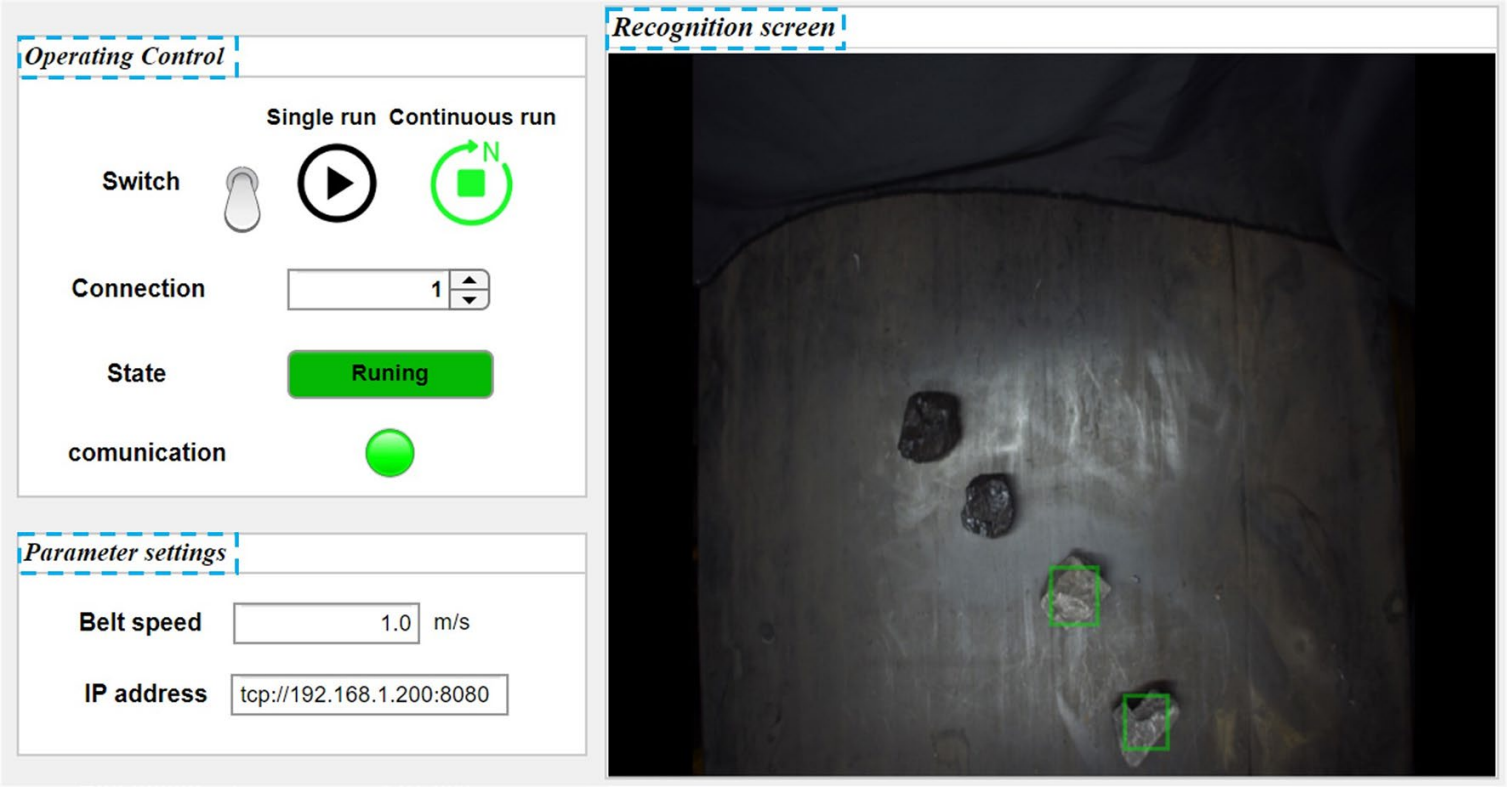
Human–computer interaction interface.

This study employs the MERLIC Designer component to develop a user-friendly human–computer interaction interface. The human–computer interaction interface primarily comprises three sections, as shown in Fig. 6.

## Coal gangue sorting control system module

**Figure 7:**
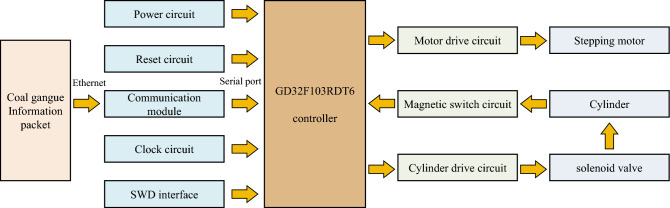
The overall design scheme of sorting module control system.

The comprehensive design framework of the sorting module control system is illustrated in Fig. 7. The coal gangue data packets are transmitted to the communication module of the sorting module control system via Ethernet. The communication module converts Ethernet data packets into serial port data packets and forwards them to the sorting module controller. The sorting module controller processes the received coal gangue identification data, regulates the corresponding cylinder actions, and completes the entire workflow of coal gangue identification and sorting. The sorting module controller adjusts the stepper motor's rotation according to the conveyor belt speed, ensuring precise impact of the coal gangue by the cylinder.

### Hardware design of sorting system

The coal gangue sorting module primarily comprises guide rails, air sources, solenoid valves, cylinders, magnetic switches, stepper motors, and stepper motor controllers. The primary technical specifications are presented in Table 4.

**Table 4:** Main technical parameters of some components of the coal gangue mold sorting module.

Device name	Model	Parameter	Numerical value
Air compressor	OTS-1100X3	Speed	1380 r/min
		Power	3.3 Kw
		Rated air pressure	0.75 Mpa
Solenoid valve	XY5220A-02-B-N-300 mm	Operating frequency	10 Hz
		Pressure	0.15–0.7 Mpa
		Voltage	DC24 V
Cylinder	SC32X150S	Inside diameter	32 mm
		Working pressure range	0.15–1.0 MPa
		Stroke	150 mm
		Compression area	804 mm^2^
Switches	CMSG-020	Rated voltage	5–240 V AC/DC
Stepping motor	56BYG250BN	Static phase current	4.0 A
		Holding torque	5.0 Nm
		Phase number	2
Stepping motor driver	SD-20504	Supply voltage	DC 24–50 V
		Output current	4.0A/phase
		Excitation method (steps/revolution)	200, 400, 800…,25,000, 25,600

**Figure 8:**
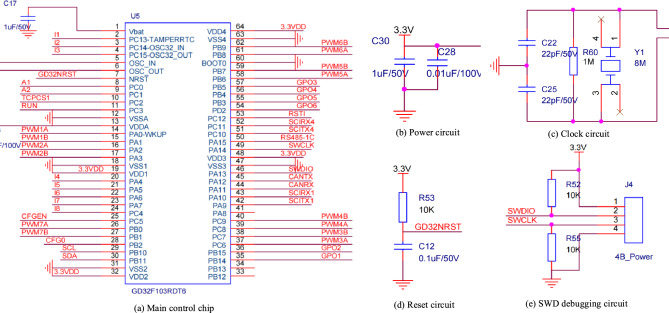
Minimum system circuit.

**Figure 9:**
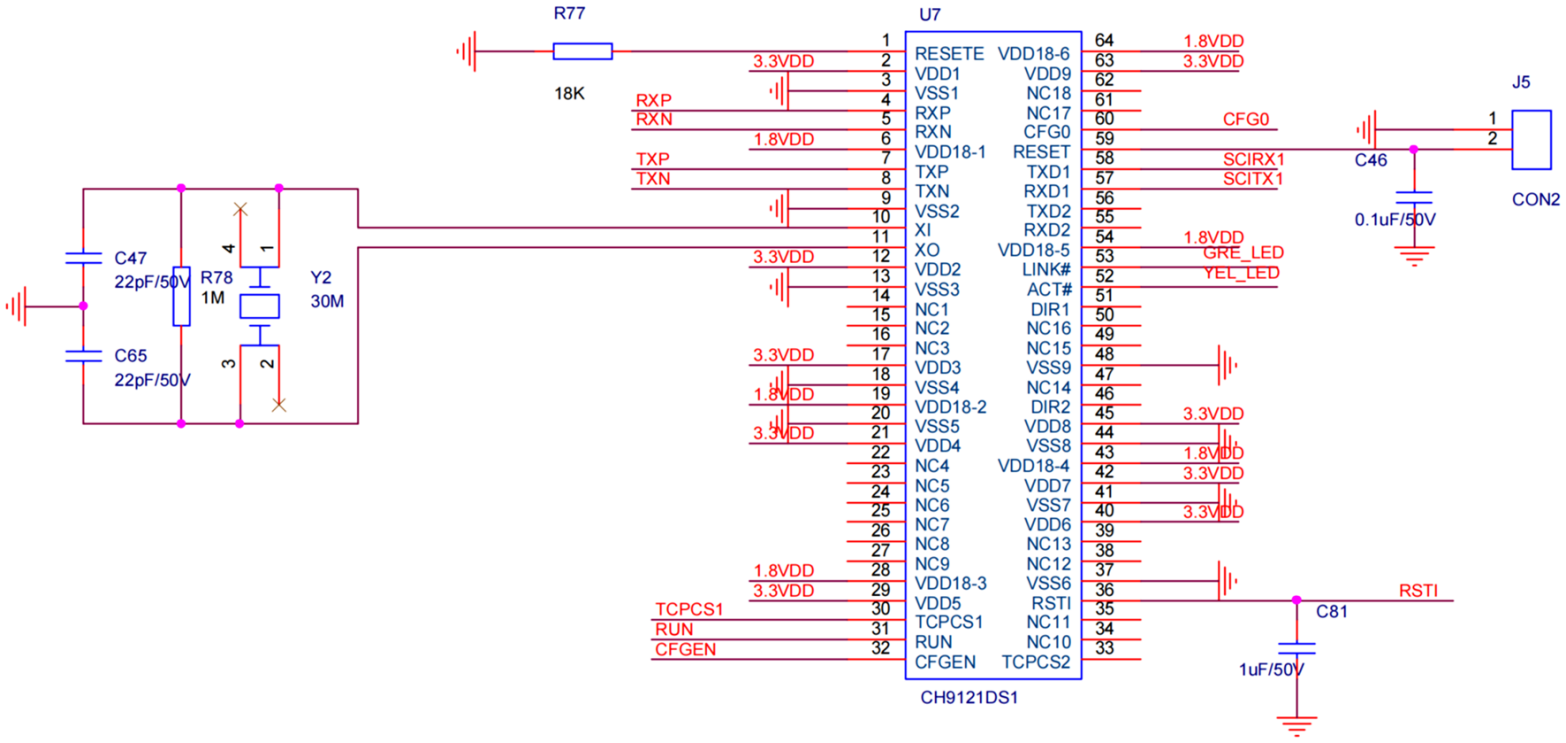
CH9121 circuit diagram.

**Figure 10:**
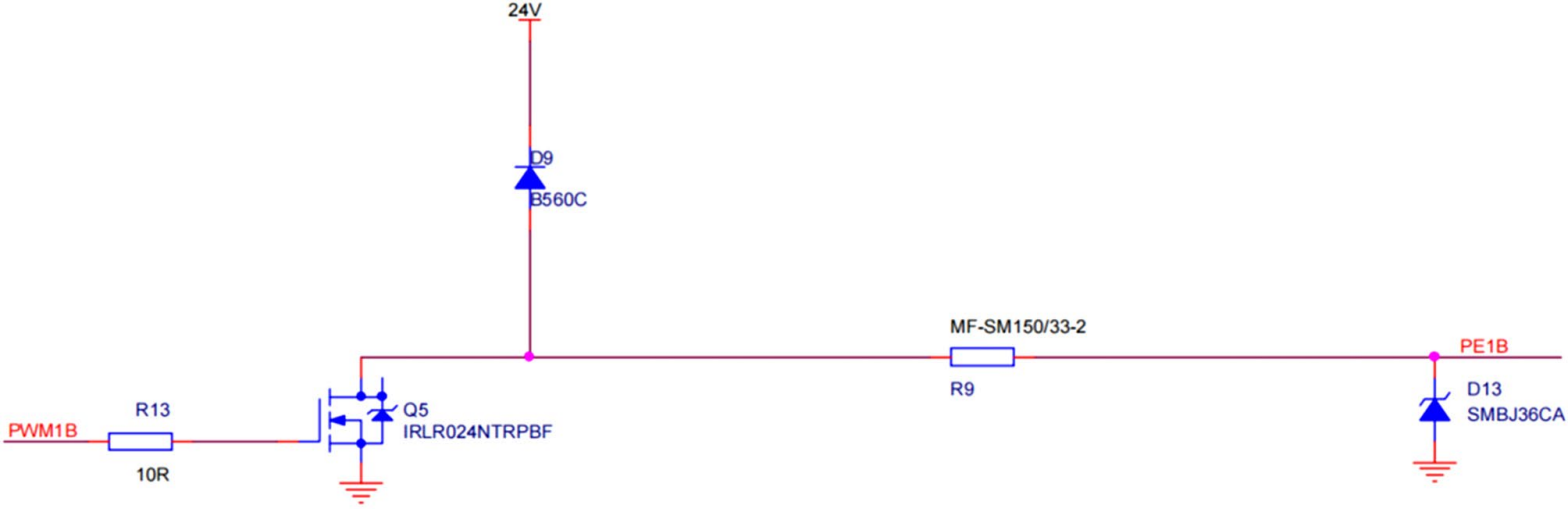
One-way cylinder drive circuit.

(1) The core circuit of the main control chip involves selecting the GD32F103RDT6 chip. The minimal system circuit includes a power supply circuit, clock circuit, reset circuit, SWD debugging circuit, etc. The circuit diagram is illustrated in Fig. 8.

(2) Communication module circuit. The identification data packets transmitted by the coal gangue recognition module are conveyed via Ethernet. The sorting controller must be equipped with compatible communication modules to receive the data packets. This study employs the CH9121 network serial communication chip to construct the communication module for the sorting control system, as illustrated in Fig. 9.

(3) The cylinder control circuit employs the XY60620A-02-B-N-300 mm two-position five-way dual electronic control solenoid valve to regulate the cylinder. Figure 10. illustrates the cylinder-driven circuit. The dual electronic control solenoid valve contains two coils: one for positive action and one for reverse action.

In the sorting system described herein, the cylinder's impact state is configured for positive action, with resetting set to reverse action.

**Figure 11:**
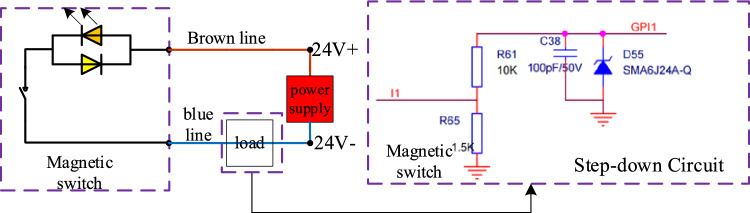
Working principle diagram of magnetic switch.

The SC32X150S model cylinder employed in this system incorporates a magnetic ring within the piston. Consequently, this system employs the CMSG-020 magnetic switch, which is compatible with the cylinder, to detect the position of the cylinder piston. The operational principle diagram of the magnetic switch is illustrated in Fig. 11. The load module depicted in the figure is designed as a step-down circuit.

**Figure 12:**
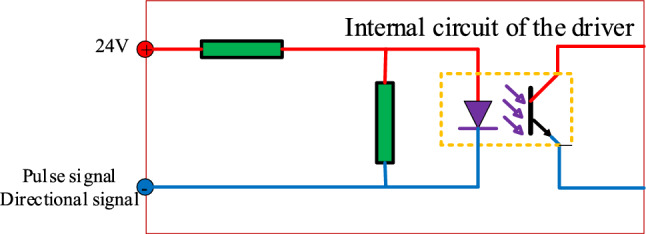
Driver input interface circuit.

(4) The sorting mechanism of the cylinder control circuit should employ a stepper motor to adjust the distance between the cylinder and the belt conveyor according to the conveyor's belt speed. This adjustment ensures that the cylinder can accurately strike the falling coal gangue within the piston's stroke range. This study employs the SD-20504 digital stepper motor driver to operate the 56BYG250BN stepper motor. The input interface circuit is illustrated in Fig. 12. The input control signal utilizes a common anode interface configuration, and the driver port is equipped with an optocoupler, which operates effectively at low levels. For pulse signal input, when the optocoupler corresponding to the pulse input port of the driver conducts, the driver receives a pulse signal and advances the motor by one step.

### Sorting system software design

During the coal gangue sorting experiment, the actual transportation capacity of the belt was constrained, and no instances of belt breakage or bulging were observed. This study employs the GD32F103RDT6 chip as the control unit, and the software design is implemented based on the hardware circuitry.

**Figure 13:**
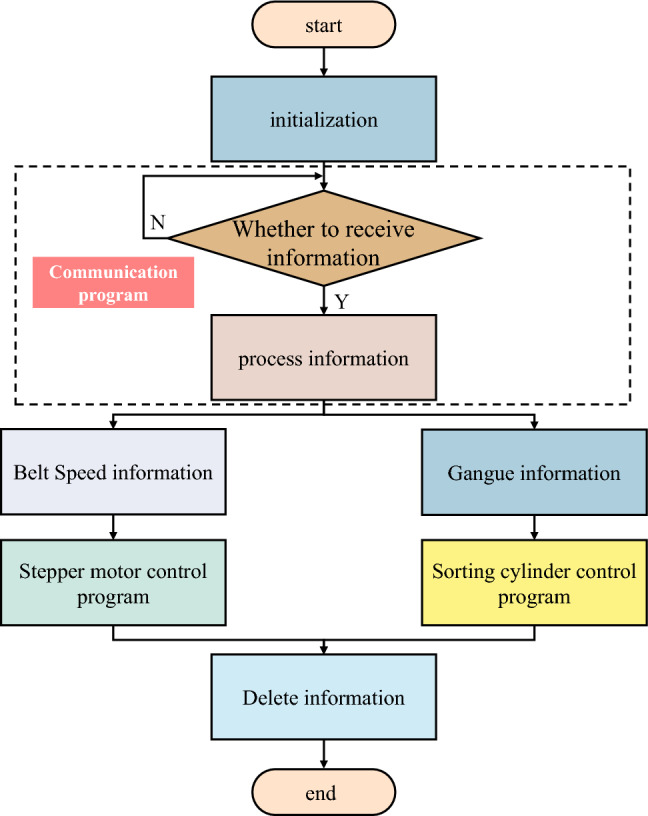
Main program flowchart.

**Figure 14:**
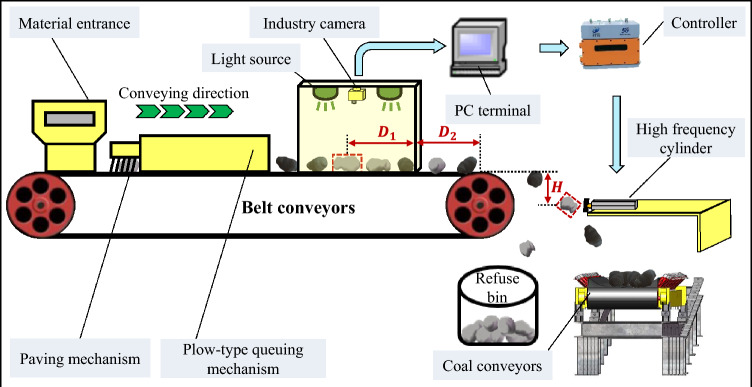
Schematic diagram of coal gangue striking.

1. Main Program Design. The primary function of the main program in the sorting module control system is to process the coal gangue identification data packets transmitted by the coal gangue identification module, in accordance with a custom communication protocol. It additionally controls the sorting actions of the cylinder or the rotation of the stepper motor based on the processed data. The flowchart of the main program design is depicted in Fig. 13.
2. Cylinder Control Program Design. The cylinder control program primarily accomplishes two tasks. First, the delay time for striking is determined based on the belt speed information, model recognition time, and Y-axis coordinate of the coal gangue obtained from the communication program. Second, the corresponding cylinder is controlled to strike after a delay time based on the X-axis coordinate information of the coal gangue obtained from the communication program. The schematic diagram of coal gangue impact is illustrated in Fig. 14.
3. The effective implementation of the project's outcomes in the primary underground transportation system will significantly enhance the raw coal selection efficiency, thereby decreasing the labor intensity of the workforce. In addition to ensuring the sorting efficiency, it also minimizes electrical energy consumption, indirectly reducing the combustion of standard coal. Consequently, this leads to a significant decrease in the emission of pollutants such as carbon dust, carbon dioxide (CO_2_), sulfur dioxide (SO_2_), and nitrogen oxides (NO_x_), thereby effectively safeguarding the ecological environment.

Figure 14 illustrates the distance between the coal gangue and the photography area, the travel distance of the coal gangue on the belt conveyor, and the height from which the coal gangue falls. The formula for calculating the strike delay time is presented in Eq. (1).

1 $$t = \frac{(1280 - y)*0.0667}{v} + \frac{{D_{2} }}{v} + \sqrt{\frac{2H}{g}} - T$$

The formula incorporates the time taken for the model to recognize images, the speed of the belt conveyor, gravitational acceleration, and fixed values of 40 cm and 63.5 cm. The dimensions of the coal gangue image are 1280 × 1280 pixels, with each pixel measuring 0.0667 cm.

(4) Stepper Motor Program Design. This study specifies a pulse frequency of 200 Hz, a duty cycle of 50%, and sets the excitation method to 200 steps per revolution. The circumference of the shaft gear on the stepper motor within the sorting system designed in this study is 8 cm. This indicates that when the motor completes one rotation, the sorting device moves linearly by 8 cm. The calculation formula for motor control is presented in Eqs. (2).

**Figure 15:**
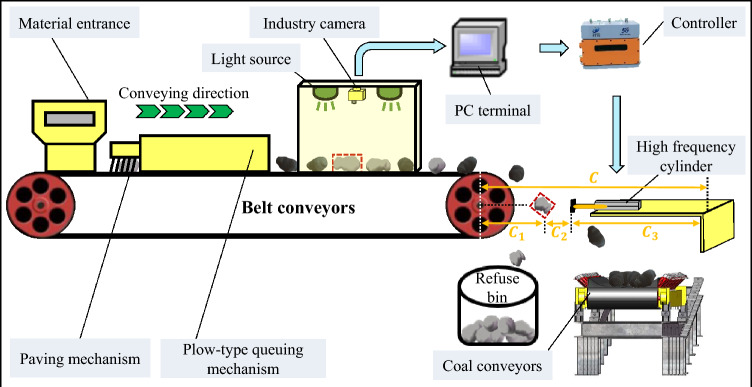
Schematic diagram of striking distance of sorting mechanism.

Figure 15 shows a schematic diagram of the sorting mechanism's impact distance. The figure depicts a fixed value of 120 cm, representing the current position of the stepper motor and the horizontal displacement during the coal gangue's descent.

2 $$m = \frac{{C - C_{3} - v\sqrt{\frac{2H}{g}} }}{s}$$

In the formula, "s" denotes the linear distance the sorting device moves after one full rotation of the motor, and "m" denotes the number of pulses.

## Experimental study on the complete machine of coal gangue sorting system

**Figure 16:**
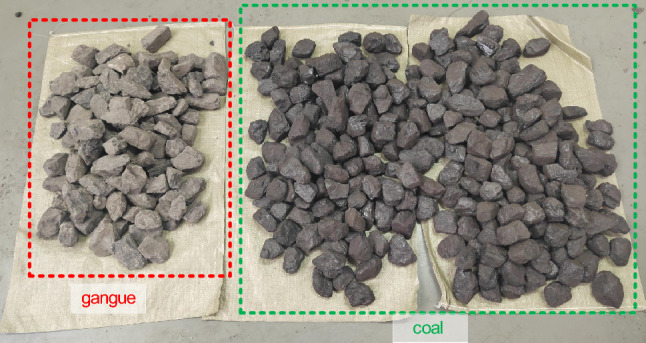
Experimental coal gangue and coal.

**Figure 17:**
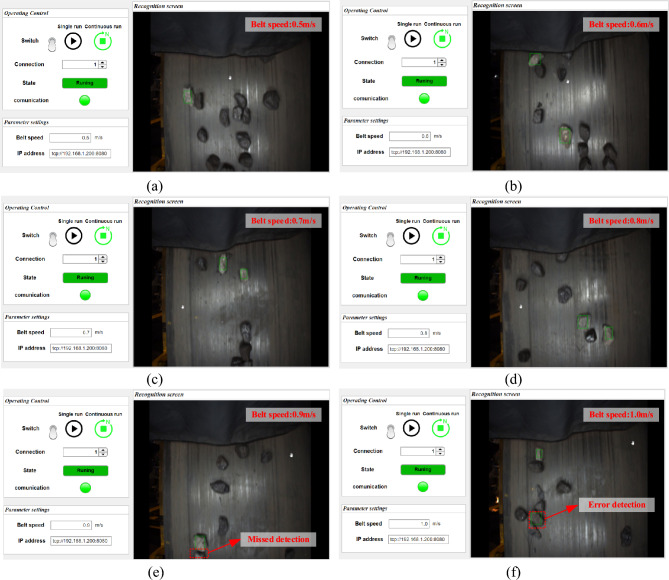
Dynamic experiment host computer recognition interface.

This study experimentally evaluates the coal gangue sorting system prototype at various belt speeds by feeding coal gangue and coal through the feeding port. After passing through the coal gangue identification and sorting modules, the prototype assesses the recognition and sorting performance of the coal flow with a gangue content of 30% at different belt speeds. The particle size of coal gangue utilized in the experiment ranges from 50 to 120 mm. Preliminary experiments indicate that an excessive particle size of coal gangue can result in insufficient cylinder force for effective separation, whereas a smaller particle size may cause the cylinder to fracture the gangue. Moreover, the particle size of coal gangue within the range of 50–120 mm is more suitable for the operational conditions of coal washing plants. The coal and coal gangue used in the experiment are illustrated in Fig. 16. The interface of the host computer used to identify the coal gangue during the dynamic experiment is shown in Fig. 17.

**Figure 18:**
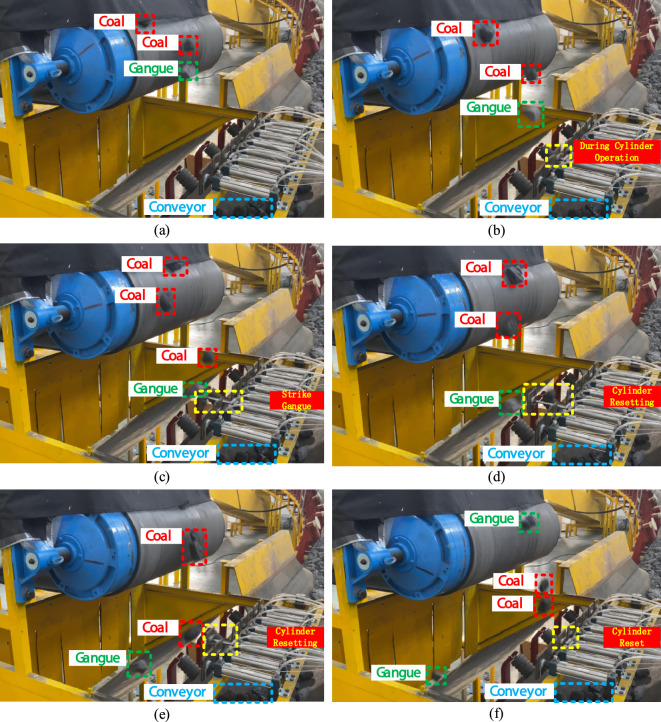
Process diagram of striking coal gangue.

Figure 18 illustrates the cylinder striking the gangue during the experiment, causing it to fall. Before the gangue reaches the cutting position, the cylinder at the corresponding location moves in advance. The gangue passes through the cylinder's impact area and is struck. The gangue deviates from its original trajectory and falls into the waste bin. The cylinder then resets. The coal smoothly passes through the cylinder's impact area and then falls onto the conveyor. The cylinder is reset. Through multiple experiments, the identification and sorting data of the prototype for coal flow with 120 coal gangue samples, 280 coal samples, and 30% gangue content at different belt speeds are presented in Table 5.

**Table 5:** Sorting experimental data.

Belt speed (m/s)	Number	Correct number	Error number		Number of sorting	Accuracy (%)	Recall (%)	Sorting rate (%)	Error rate (%)
0.5	119	118		1	118	99.2	98.3	98.3	1.7
0.6	118	117		1	117	99.2	97.5	97.5	2.5
0.7	118	116		2	115	98.3	96.7	95.8	4.2
0.8	116	115		1	114	99.1	95.8	95.5	4.5
0.9	115	112		3	112	97.4	93.3	93.3	6.7
1.0	114	111		3	110	97.3	92.5	91.7	8.3

According to Table 5, the prototype of the coal gangue sorting system achieves a recognition accuracy of over 97%, a recall rate exceeding 92%, and a sorting rate surpassing 91% within the belt speed range of 0.5–1 m/s. At a speed of 0.5 m/s, the sorting system prototype achieves its highest accuracy, recall, and sorting rates, with values of 99.2%, 98.3%, and 98.3%, respectively. This demonstrates an effective separation of coal gangue. In previous studies, the author has accomplished the recognition and sorting of coal gangue utilizing image processing and multi-layer perceptron techniques^24,31^. The method employs image processing combined with multi-layer perceptron to identify coal and gangue, followed by the use of a robotic arm for sorting. The experimental results indicate that the average recognition accuracy for coal gangue is 96.45%, while the average sorting rate is 90.76%. These results are derived from the average of multiple experiments. At a belt speed of 0.6 m/s, the detailed comparison of experimental data is presented in the Table 6.

**Table 6:** Experimental comparison.

Method	Belt speed (m/s)	Number	Accuracy (%)	Recall (%)	Sorting rate (%)
This paper	0.6	118	99.2	97.5	97.5
Other^24^	0.6	200	96.9	94.3	81.5
Other^31^	0.6	200	–	–	95

The data in the table demonstrate that the coal gangue identification and sorting method employed in this study outperforms other methods and yields consistently positive results.

Regarding energy efficiency in the sorting system, this study employs a pneumatic cylinder to strike the gangue, thereby completing the sorting process. Compared to spray gun sorting and mechanical arm sorting^32,33^, this method offers several advantages.

Table 7 illustrates that different methods are employed for sorting coal gangue of varying sizes. When sorting coal gangue of similar sizes, the cylinder-type sorting method utilized in this study consumes less energy, involves a simpler process, and provides superior application benefits.

**Table 7:** Comparison of energy consumption.

Method	Power	Exhaust volume	Gangue radius	Load weight	Degree of freedom
This paper	3.3 Kw	0.75 MPa	50–120 mm	3 kg	–
Other^32^	–	0.75–1.30 MPa	50–300 mm	–	–
Other^33^	10Kw	–	4–200 mm	4 kg	6

## Conclusion

Addressing the issues of low recognition accuracy, inefficiency, and resource wastage inherent in traditional coal selection technologies at coal washing plants, a pneumatic intelligent coal gangue sorting system based on deep learning is proposed. This system effectively enhances the inclusion rate of raw coal. The main conclusions of this study are as follows:

1. Based on the practical application scenarios of coal washing plants, a coal gangue sorting system capable of accurately identifying and sorting coal gangue with particle sizes ranging from 50 to 120 mm is proposed. The hardware selection and software design for the relevant modules, including the belt conveyor module, paving and queuing module, coal gangue identification module, and coal gangue sorting module, have been completed.
2. To address the low accuracy in coal and gangue recognition and poor positioning precision, this study employs the ResNet-50 network model, which demonstrates optimal performance within the HALCON software, as the gangue recognition module. Compared to most algorithms, this model exhibits higher accuracy and superior dynamic adaptability for coal gangue recognition, achieving recognition accuracy exceeding 97% within a belt running speed range of 0.5 to 1.0 m/s.
3. To address the inefficiencies and high energy consumption of existing gangue selection technologies, a pneumatic ejection sorting device based on multi-objective dynamic identification was developed. Compared to technologies such as multi-axis mechanical grippers and dense medium gangue separation, this system achieves a coal and gangue sorting rate exceeding 91%, with lower production costs, reduced energy consumption, and a separation completion time of less than 3 s.
4. Utilizing the designed prototype platform of the coal gangue separation system, experiments were conducted on coal flow containing 30% gangue. The results indicate that, compared to existing coal gangue separation technologies, this system simplifies the process flow, significantly improves the coal gangue sorting rate, and features lower energy consumption and response time.

## Data Availability

Due to the ongoing nature of the project, the datasets obtained during the current study are temporarily not publicly available but can be obtained from the corresponding authors upon reasonable request.
